# PICU Face and Thoracoabdominal Detection Using Self-Supervised Divided Space–Time Mamba

**DOI:** 10.3390/life15111706

**Published:** 2025-11-04

**Authors:** Mohamed Khalil Ben Salah, Philippe Jouvet, Rita Noumeir

**Affiliations:** 1 Biomedical Information Processing Laboratory, École de Technologie Supérieure, University of Quebec, Montreal, QC H3C 1K3, Canada; rita.noumeir@etsmtl.ca; 2 Pediatric Intensive Care Unit, CHU Sainte-Justine, Montreal, QC H3T 1C5, Canada; philippe.jouvet.med@ssss.gouv.qc.ca

**Keywords:** self-supervised learning, state space models, non-contact vital sign monitoring, PICU, multimodal RGB-D

## Abstract

Non-contact vital sign monitoring in Pediatric Intensive Care Units is challenged by frequent occlusions, data scarcity, and the need for temporally stable anatomical tracking to extract reliable physiological signals. Traditional detectors produce unstable tracking, while video transformers are too computationally intensive for deployment on resource-limited clinical hardware. We introduce Divided Space–Time Mamba, an architecture that decouples spatial and temporal feature learning using State Space Models to achieve linear-time complexity, over 92% lower than standard transformers. To handle data scarcity, we employ self-supervised pre-training with masked autoencoders on over 50 k domain-specific video clips and further enhance robustness with multimodal RGB-D input. Our model demonstrates superior performance, achieving 0.96 mAP@0.5, 0.62 mAP50-95, and 0.95 rotated IoU. Operating at 23 FPS (43 ms latency), our method is approximately 1.9× faster than VideoMAE and 5.7× faster than frame-wise YOLOv8, demonstrating its suitability for real-time clinical monitoring.

## 1. Introduction

Monitoring vital signs in pediatric patients within Pediatric Intensive Care Units (PICUs) is essential due to their fragile health conditions. Non-contact approaches, such as remote photoplethysmography (rPPG) and respiratory monitoring, especially for conditions like Acute Respiratory Distress Syndrome (ARDS) [1,2], depend on accurate detection of anatomical regions, particularly the face and thoracoabdominal areas. Accurate and anatomically consistent localization is essential for reliable vital sign estimation in PICU environments. Remote photoplethysmography (rPPG) depends on stable face crops that preserve skin-only regions, as chrominance fluctuations are easily corrupted by background leakage or bounding box drift. Even minor spatial jitter or rotation error can distort the rPPG signal, degrading heart rate accuracy and introducing artifacts into the frequency spectrum. Similarly, thoracoabdominal detection must capture the cyclic expansion of the chest along the correct orientation axis to extract respiratory motion cues. Boxes that drift or encompass non-thoracic regions, such as blankets or bedrails, risk obscuring the subtle periodic deformations that encode respiration.

The PICU setting presents several challenges: patients are frequently obscured by medical devices, lighting conditions, and patient orientations, and there is a lack of annotated data necessary for training effective models for our specific clinical setting. Notably, no existing public video datasets capture both facial and thoracoabdominal regions simultaneously in PICU environments, necessitating the creation of our own dataset. In addition to RGB inputs, depth information provides complementary geometric context that improves robustness to occlusions and illumination variability, both of which are frequent in PICU environments. Unlike RGB, depth is invariant to lighting changes and can help distinguish foreground anatomical structures from background clutter such as tubing, blankets, or bedrails. This is particularly valuable when visual cues are weak or partially obstructed. In such cases, depth enhances the stability of region tracking and supports more reliable detection of subtle motion patterns. However, consistent acquisition and integration of depth data in real-world clinical settings remain non-trivial, requiring careful sensor placement, calibration, and synchronization with RGB streams to ensure reliable performance across patient conditions and hardware setups.

Data scarcity remains one of the main challenges in the medical domain, primarily due to strict privacy constraints and ethical considerations. Collecting large-scale, manually annotated video datasets in a clinical environment like the PICU is exceptionally difficult and costly. Furthermore, there is a significant domain gap between general-purpose videos and clinical data; features learned from datasets such as Kinetics-400 often fail to generalize to the unique PICU setting [3], limiting model performance across different healthcare settings [4,5]. Downstream tasks in the PICU are especially challenging due to the subtle motion patterns and unique anatomical features of pediatric patients, where standard face detectors often fail because of underdeveloped facial structures and frequent occlusions from medical equipment [6,7]. To address both data limitations and domain discrepancies, self-supervised learning (SSL) provides a promising solution. Pre-training on unlabeled hospital videos allows models to learn relevant spatiotemporal features directly from the target environment, reducing dependence on manual annotations [8]. Among SSL methods, masked autoencoders such as VideoMAE [9] have demonstrated strong efficiency, offering robust representation learning for clinical settings where annotated data is limited.

While convolutional neural networks (CNNs) have significantly advanced static object detection and tracking, with strong performance on large-scale datasets such as COCO [10] and PASCAL VOC [11], these models are not designed to capture the temporal dynamics essential for physiological monitoring [12]. Reliable signal monitoring depends on stable region-of-interest (ROI) tracking across time [1,13]. Frame-based detectors, including recent YOLO variants, are limited in this context for two main reasons: first, they often suffer from temporal inconsistencies such as bounding box jitter, which introduces motion artifacts into the predicted signals [14,15]; second, their per-frame inference leads to high computational overhead, making them less suitable for real-time applications.

The Video Vision Transformers (ViViT) model [16,17,18] relies on self-attention mechanisms to capture long-range dependencies and global context. However, it is computationally expensive and often struggles to generalize, especially when trained on limited datasets. Its lack of inherent inductive biases, such as spatial locality, makes it less effective in data-constrained environments. Moreover, its resource requirements increase quadratically when processing high-resolution inputs or integrating multimodal data like RGB and depth, which makes it difficult to deploy in real-world clinical settings. In contrast, 3D convolutional neural networks (3D-CNNs) [19,20] possess strong inductive biases, making them more suitable for limited-data scenarios. However, their localized kernel limits their ability to model long-range spatiotemporal dependencies, which are crucial for understanding complex clinical settings.

PICU monitoring systems must process multiple patient video streams continuously on shared, resource-limited workstations, often without dedicated accelerators or cloud offloading due to privacy and maintenance constraints. Reliable bedside use therefore requires low-jitter, real-time inference with a small memory footprint while coexisting with other clinical software. Under these conditions, models whose time and memory scale quadratically with the token length *L* (e.g., multi-head self-attention, $O(L^{2})$) become impractical as resolution or temporal context grows. This motivates architectures with linear-time complexity (O(L)) and low VRAM that maintain stable latency on longer clips and higher resolutions.

Reliable face and thoracoabdominal localization is a prerequisite for contactless vital-sign monitoring in the PICU. The face region provides the skin pixels needed for rPPG, where minute chrominance fluctuations encode heart rate; any drift or background leakage rapidly degrades signal-to-noise and produces unstable frequency peaks. The thoracoabdominal region carries respiratory motion, so bounding boxes must remain temporally stable and orientation-aware to capture cyclic expansion/deflation without being contaminated by blankets, caregiver hands, or attached devices. Axis-aligned boxes are often insufficient under infant pose changes, bed tilt, and off-axis cameras; oriented bounding boxes (OBBs) yield tighter, rotation-consistent crops, improving both rPPG sampling using skin-only pixels and respiration estimation with motion along the anteroposterior axis. These constraints, coupled with frequent occlusions, specular lighting, and the need for real-time processing on clinical hardware, motivate a detector that is robust, temporally stable, and computationally efficient for face and thoracoabdominal ROIs, precisely the focus of our approach below.

To address these challenges, we introduce Divided Space–Time (DST) Mamba, an SSM-based detector for face and thoracoabdominal regions with oriented bounding boxes (OBBs). The model is built on Selective State Space Models (SSMs) [21] and runs in linear time O(L) with respect to sequence length *L*, which is essential for real-time monitoring. Unlike VideoMamba, which processes space and time jointly, DST decouples them: a spatial stage followed by a temporal stage. This factorization reduces cross-axis interference, preserves temporal dynamics relevant to rPPG/respiration, and enables axis-specific optimization. It also allows independent MAE pre-training for spatial masked patches and temporal masked tube objectives. To handle PICU conditions, like occlusions and low contrast, we use data-efficient masked-autoencoding pre-training and support multimodal input (RGB + depth) to improve perceptual robustness.

The primary contributions of this work are outlined as follows:

- Reliable ROI detection in PICU videos: we successfully detect the face and thoracoabdominal regions with oriented boxes despite occlusions, devices, motion, and limited labels in the PICU. This robustness is achieved via a Divided Space–Time Mamba design that preserves temporal dynamics while remaining computationally efficient.
- Data scarcity and domain gaps addressed: we mitigate scarce annotations and lab-to-PICU domain shift by employing self-supervised masked-autoencoder pre-training tailored to our clinical video distribution.
- Multimodal robustness under occlusion: we enhance detection accuracy and reduce angle drift in occlusion-heavy scenes by integrating RGB with depth and analyzing the accuracy–complexity trade-off for deployment.
- Clinical-grade efficiency and comparative gains: we achieve real-time throughput and low FLOPs while outperforming strong frame-wise and video baselines on accuracy and temporal stability through a factorized spatial-to-temporal SSM pipeline and targeted ablations.

## 2. Related Works

### 2.1. Object Detection in Videos

Deep learning techniques have significantly advanced object detection. Initially, two-stage detectors like R-CNN [22], Fast R-CNN [23] and Faster R-CNN [24] utilized region proposals to achieve high accuracy. Single-stage detectors, including the YOLO family of models [25,26,27] and the Single Shot Multibox Detector (SSD) [28], and highly efficient architectures like EfficientDet [29], emerged as faster alternatives by predicting bounding boxes and class probabilities in a single pass. More recently, transformer-based approaches such as DETR [30], Deformable DETR [31], and Vision Transformer Detector (ViTDet) [32] have been proposed, leveraging global context through Multi-Head Self-Attention to enable end-to-end object detection.

However, these methods process frames independently, relying on separate tracking modules (e.g., YOLO + DeepSORT [14]) for temporal coherence, which increases latency and introduces jitter or artifacts that degrade vital sign signals in critical care [33,34]. This gap highlights the importance of using factorized spatiotemporal modeling to maintain temporal stability and orientation-aware detection under real-time constraints. By avoiding reliance on external trackers and ensuring linear complexity, such designs support consistent performance in PICU monitoring scenarios.

### 2.2. Face Detection in Complex Environments: NICU/PICU

Face detection in NICU/PICU settings has evolved from traditional hand-crafted methods like Haar cascades with AdaBoost [35] to deep learning models such as MTCNN [36], RetinaFace [37], BlazeFace [38], and YOLO5Face [39], which offer robustness to occlusions and real-time performance.

Standard detectors struggle with neonatal morphology, medical occlusions, and cluttered backgrounds, as shown in NICU-specific adaptations such as NICU-Face (YOLOv5-based) [7], Hausmann’s model [40], and Grooby’s YOLOv7 [41]. Integrated approaches for vital sign estimation, including Huang et al. [42] for heart rate and Kyrollos et al. [43] for respiration, still lack temporal consistency, often resulting in unstable region tracking and noisy physiological signals. In contrast, our approach leverages self-supervised pre-training on domain-specific video data and incorporates multimodal RGB-D input to improve robustness to occlusions and ensure stable tracking of anatomical regions for continuous monitoring.

### 2.3. Thoracoabdominal Detection and Respiratory ROI Tracking

Thoracoabdominal detection for respiratory monitoring often relies on depth sensors and classical approaches, including infrared imaging for torso motion [44], time-of-flight cameras with anatomical landmarks [45], and segmentation-based techniques such as normalized cuts [46] or probability masks [47]. Rehouma et al. [48] reconstructed a 3D thoracoabdominal surface using dual Kinect v2 sensors to capture respiratory patterns. Simpler pixel-tracking methods [49,50] and static deep learning models [51,52] have also been proposed, although they lack the ability to capture temporal dynamics.

Frame-based approaches fail to preserve periodic respiratory motion under occlusion, emphasizing the need for spatiotemporal continuity. The decoupled temporal design employed in our method captures subtle inter-frame motion patterns, while the use of oriented bounding boxes enables rotation-consistent localization in occlusion-heavy PICU environments.

### 2.4. Video Understanding Models

To address the need for temporal continuity, models that process video data have been developed. Three-dimensional convolutional neural networks (3D-CNNs) [19,20] extend standard CNNs into the temporal dimension by convolving across successive frames. While 3D-CNNs effectively learn short-term motion features, their fixed temporal receptive field limits their ability to capture long-range dependencies, such as full breathing cycles or prolonged occlusions. Increasing their depth or temporal window significantly raises computational costs, making them impractical for long PICU video sequences [53,54,55].

More recent transformer-based video models, such as ViViT [16] and TimeSformer [17], apply self-attention to sequences of frame patches, effectively modeling global spatiotemporal relationships. These approaches achieve strong performance on action recognition benchmarks by capturing interactions across entire clips. However, their self-attention mechanism has quadratic complexity with respect to the number of tokens (spatial patches × temporal frames), resulting in high memory and computational demands. For example, processing a 30-s PICU video at clinically meaningful resolution would involve attending over a high-dimensional token sequence, making such models impractical for real-time deployment without specialized hardware.

### 2.5. Self-Supervised Video Representation Learning

In data-limited environments, self-supervised learning (SSL) offers an efficient strategy for pre-training models on unlabeled videos by constructing surrogate tasks. This is particularly relevant in medical contexts, where data collection is constrained by privacy concerns and manual annotation is costly [8]. By learning directly from the data, SSL enables models to acquire meaningful representations that can be transferred to downstream tasks such as detection or segmentation. A common SSL approach for video is contrastive learning, where models are trained to map different augmentations of the same video clip to similar embeddings, while pushing apart embeddings from different clips. Momentum Contrast (MoCo) [56] and SimCLR [57] are prominent examples using instance discrimination objectives. More recent variants such as BYOL [58] and DINO [59] eliminate the need for explicit negative samples by employing teacher–student architectures to learn invariant features from augmented video data.

Another class of self-supervised learning (SSL) methods is generative or reconstruction-based. These approaches involve masking or removing parts of the input and training the model to predict the missing content, thereby encouraging it to learn contextual and semantic structures. Masked image modeling (MIM) techniques, inspired by BERT in natural language processing (NLP) [60], have shown strong performance in both image and video domains. For example, iGPT [61] and BEiT [62] demonstrated the effectiveness of tokenizing images and learning through masked token prediction. In particular, Masked Autoencoders (MAE) [63] showed that a vision transformer can be pre-trained efficiently by encoding only a small subset of visible image patches and training a lightweight decoder to reconstruct the missing ones. VideoMAE [9] extended this concept to video by leveraging the high redundancy between frames. It employs an extremely high masking ratio (90–95%) using a tube masking strategy, which masks consistent spatial regions across consecutive frames, enabling efficient learning of spatiotemporal representations. A common challenge with reconstruction-based self-supervised learning (SSL) is that optimizing for low-level pixel accuracy may not produce representations that capture high-level semantic features. Recent advancements, such as Unmasked Teacher (UMT) [64], address this limitation by incorporating a teacher network that identifies informative tokens and provides softer reconstruction targets, thereby guiding masked autoencoders toward learning more semantic representations.

Nonetheless, a key advantage of the MAE approach in our context is that it enables pre-training directly on our collected PICU video data, as well as on additional video sequences created from real clinical images captured in PICU/NICU settings, without requiring any labels. This allows the model to learn representations adapted to the hospital environment, such as the appearance of neonatal skin under PICU lighting or the typical motion patterns of breathing infants, effectively bridging the domain gap encountered when using models pre-trained on general-purpose video datasets such as Kinetics-400.

### 2.6. State Space Models (SSMs)

Transformers have become the dominant architecture for sequence modeling in both natural language processing (NLP) and computer vision due to their capacity for global attention [65]. However, their $O(n^{2})$ complexity with respect to sequence length makes them less tractable for very long sequences or high-resolution video. State Space Models (SSMs) offer an alternative sequence modeling paradigm with O(n) complexity, based on simulating linear dynamical systems. The Structured State Space Sequence Model (S4) [66] introduced a parameterization that enables learning long-range dependencies via a diagonal-plus-low-rank representation of the state transition matrix, achieving strong performance on long-sequence tasks while maintaining linear time complexity. Subsequent refinements, including S5 [67], H3 [68], and GSS [69], further improved the stability and efficiency of SSM-based sequence layers.

Mamba [21], based on the State Space Model (SSM), introduced a data-dependent SSM layer that enables efficient processing of long sequences while maintaining computational efficiency. It incorporates Selective State Spaces by making the state transition matrices input-dependent, allowing the model to selectively process information based on the current input. In addition, it employs hardware-aware parallelism to optimize long-sequence processing by avoiding unnecessary memory access through selective scans and kernel fusion. This design minimizes latency, maximizes throughput on modern GPUs, and achieves true linear-time scaling. Mamba outperforms Transformer architectures on large-scale real-world datasets and scales linearly with sequence length. Recently, several Mamba-based approaches have leveraged the strengths of SSMs to efficiently model long sequences [70,71].

The Mamba architecture has been extended to computer vision tasks through several adaptations. Vision Mamba (ViM) [72] generalizes Mamba from 1D to 2D sequences by employing bidirectional scans, processing all tokens in both forward and backward directions to enhance spatial representations. VMamba [73] introduces a different scanning strategy using 2D Selective Scan (SS2D), which processes tokens in four directions to enrich contextual information. EfficientVMamba [74] proposes a lightweight model that applies atrous sampling on feature map patches to reduce computational complexity. To improve local representation, LocalMamba [75] divides the image into groups and scans each group’s window independently. It also incorporates spatial and channel attention modules to filter out redundant information and retain only the most relevant features. The strong performance of Mamba-based backbones across diverse vision tasks has spurred the development of specialized models tailored to specific applications [76,77,78,79,80].

3D convolutional neural networks (3D CNNs) are computationally expensive and memory-intensive, while video transformers suffer from poor scalability due to the quadratic complexity of self-attention with respect to input length. Both approaches tend to be slow, resource-demanding, and require substantial amounts of training data. To address these limitations, Mamba has recently been extended to video understanding tasks, offering a more efficient alternative to 3D CNNs and video transformers. VideoMamba [81] is designed to maintain linear complexity for long-range video modeling. It begins by dividing the input video into non-overlapping spatiotemporal patches using a 3D CNN, followed by the addition of learnable spatial and temporal positional embeddings. Spatial tokens are arranged according to their locations and stacked sequentially across frames. Leveraging Mamba’s linear-time Selective State Space mechanism, VideoMamba can efficiently process long, high-resolution video sequences. However, due to the phenomenon of historical decay, where earlier tokens have limited influence on later outputs; VideoMambaPro [82] improves upon the original model by introducing masked backward computation in the bidirectional Mamba process and residual connections within the Mamba transition matrices.

In contrast, we propose a Divided Space–Time Mamba architecture that explicitly decouples spatial and temporal sequence modeling. Inspired by the factorized space-time attention in TimeSformer [17], our model processes spatial and temporal information in separate stages: spatial Mamba layers first operate within each frame to preserve high-resolution spatial details, followed by temporal Mamba layers that model dependencies across frames using the spatially encoded features. The factorized space–time design enables the model to develop specialized representations along each axis, preserving high-resolution anatomical detail through spatial encoding while capturing temporal dynamics. By decoupling spatial and temporal processing, the architecture avoids the representational and computational trade-offs inherent in joint spatiotemporal models. This separation prevents subtle, time-sensitive signals from being overwhelmed by dominant spatial features, a limitation often observed in unified attention-based approaches.

## 3. Proposed Method

### 3.1. Overview

**Figure 1:**
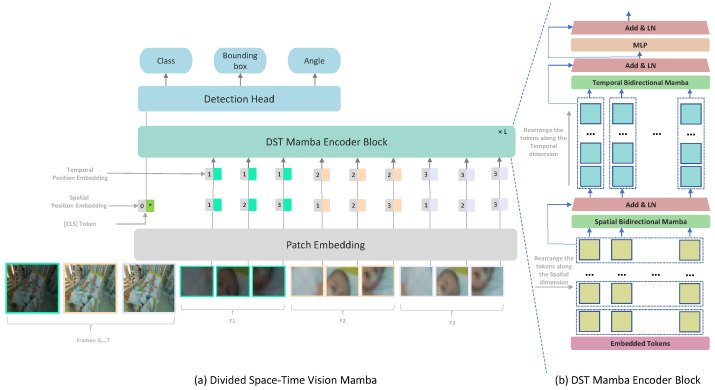
Overview of our framework: (**a**) Divided Space–Time Video Mamba integrates patch embedding, positional embeddings, Mamba encoder layers, and a detection head for class labels, bounding boxes, and angles. (**b**) The DST Mamba Block. Input tokens are first processed by a spatial Mamba layer that operates within each individual frame. The output is then reshaped and processed by a temporal B-Mamba layer across all frames. * denotes the CLS token position.

In this work, we propose a Mamba-based approach for medical video detection that reliably localizes the face and thoracoabdominal regions in PICU videos. The backbone factorizes spatiotemporal modeling by stacking Divided Space–Time (DST) Mamba blocks, which first consolidate spatial structure before modeling temporal dynamics. This sequential design allows the encoder to capture both coarse scene layouts and fine-grained motion cues, all while preserving temporal signals with linear-time complexity. On these features, a lightweight Mamba-based detection head predicts oriented bounding boxes (OBBs), yielding rotation-consistent localization under pose changes, bed tilt, and off-axis cameras, as shown in Figure 1. To address data scarcity and domain shift, we investigate two SSL pre-training techniques: Masked Autoencoders (MAE) and Unmasked Teacher (UMT). MAE reconstructs masked patches to learn robust local appearance priors, whereas UMT distills higher-level spatiotemporal structure from a teacher without masking. We quantify the contribution of SSL and depth by comparing VideoMamba and our DST-Mamba with and without pretraining and with RGB versus RGB-D input. All models are first pretrained on a combined set of CHU Sainte-Justine PICU clips and publicly available pediatric data, then fine-tuned on the PICU dataset for face and thoracoabdominal OBB detection. This comparative design targets the core clinical video challenges of data scarcity, heavy occlusions, and rapid domain shift, while favoring deployment through linear-time inference.

### 3.2. Preliminary Explanation: State Space Models

State Space Models (SSMs) map a 1-D function or sequence x(t)∈R↦y(t) using a hidden state $h\left(t\right)\in R^{N}$. This system is described as linear ordinary differential equations (ODEs), employing matrices $A\in R^{N\times N}$ to define how the hidden state evolves and $B\in R^{N\times 1}$ and $C\in R^{1\times N}$ for the projection of the input and the hidden state to the output:

(1) $$\begin{array}{cc} h^{\prime }\left(t\right) & =Ah(t)+Bx(t),\\ y(t) & =Ch(t). \end{array}$$

S4 [66] and Mamba [21] integrate a timescale parameter Δ to discretize the continuous system and convert the continuous parameters A,B to discrete parameters $\bar{A},\bar{B}$. The transformation is defined as follows:

(2) $$\bar{A}=\exp\left(\Delta A\right),\;\;\bar{B}={\left(\Delta A\right)}^{-1}\left(\exp\left(\Delta A\right)-I\right)\cdot \Delta B.$$

After the discretization of $\bar{A},\bar{B}$, the (1) is transformed into:

(3) $$\begin{array}{cc} h_{t} & =\bar{A}h_{t-1}+\bar{B}x_{t},\\ y_{t} & =Ch_{t}. \end{array}$$

A global convolution is employed to compute the model output:

(4) $$\begin{array}{cc} \bar{K} & =(C\bar{B},C\bar{A}\bar{B},\ldots ,C{\bar{A}}^{M-1}\bar{B}),\\ y & =x*\bar{K}. \end{array}$$

Here *M* represents the length of the input sequence *x*, and $\bar{K}\in R^{M}$ is a structured convolutional kernel.

### 3.3. Divided Space–Time Video Mamba

#### 3.3.1. Baseline: Joint Spatiotemporal Processing

**Figure 2:**
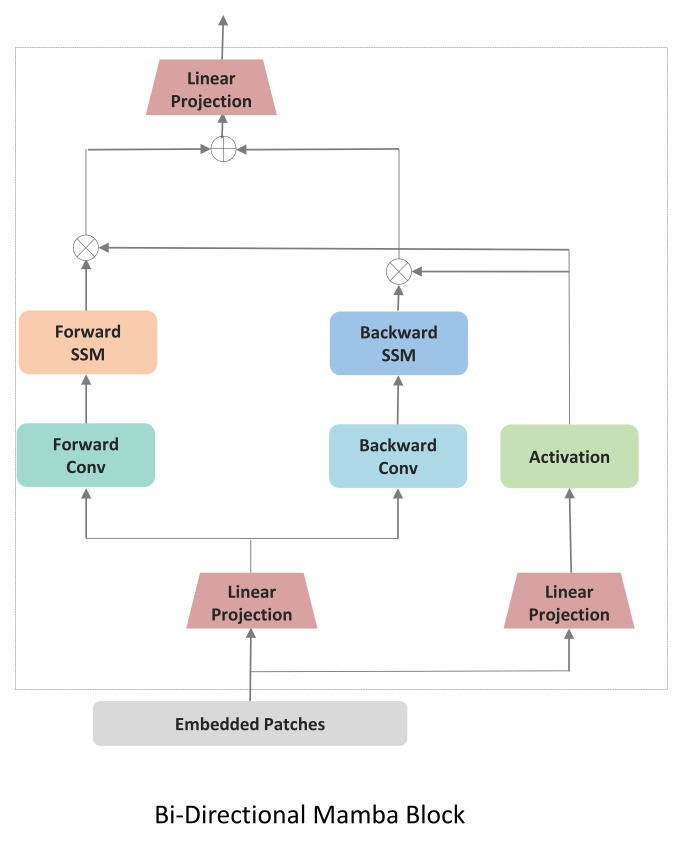
Detailed architecture of the Bidirectional Mamba block. The input sequence of Embedded Patches is processed through two parallel streams to capture dependencies in both forward and backward directions.

We first implemented a baseline following VideoMamba [81] which processes spatial and temporal information jointly through a unified bidirectional scanning mechanism, Figure 2. VideoMamba extends the Mamba state space model to video understanding by treating the entire video as a single sequence of spatiotemporal tokens. Given an input video $X\in R^{3\times T\times H\times W}$, where *T* is the number of frames and H×W are spatial dimensions, VideoMamba first applies a 3D convolutional patch embedding to obtain *N* spatiotemporal patches $X_{p}\in R^{N\times D}$; where $N=\frac{T\cdot H\cdot W}{P^{2}}$ for *P* is the patch size, and *D* is the embedding dimension. Each token represents a local spatiotemporal cube containing information from multiple consecutive frames. VideoMamba [81] applies a joint scanning strategy. All *N* tokens are arranged in a single sequence according to a spatial-first ordering:

(5) $$S_{\mathrm{joint}}=\left[x_{1,1},x_{2,1},\ldots ,x_{HW/P^{2},1},x_{1,2},\ldots ,x_{HW/P^{2},T}\right]$$

This sequence is then processed by bidirectional Mamba blocks:

(6) $$Y_{\mathrm{forward}}={\mathrm{SSM}}_{\mathrm{forward}}\left(S_{\mathrm{joint}};A,B,C,\Delta \right)$$

(7) $$Y_{\mathrm{backward}}={\mathrm{SSM}}_{\mathrm{backward}}\left(S_{\mathrm{joint}};A,B,C,\Delta \right)$$

(8) $$Y_{\mathrm{joint}}=Y_{\mathrm{forward}}+Y_{\mathrm{backward}}$$

where $A\in R^{N\times N}$, $B\in R^{N\times 1}$, $C\in R^{1\times N}$ are the state space parameters, and Δ is the time-scale parameter. The bidirectional scan enables each token to aggregate context from both past and future tokens in the sequence. All the patches are processed and then *L* Bidirectional Mamba blocks are used, where a spatial-first bidirectional scan is applied.The joint approach may not allow for fine-tuning the balance between spatial and temporal processing. By processing spatial and temporal information jointly, the model might not develop specialized features for each dimension.

#### 3.3.2. Divided Space–Time Processing

While TimeSformer [17] employs a Divided Space–Time Multi-Head Self-Attention (MHSA), its quadratic complexity with respect to token count poses challenges for long video sequences, where token numbers grow linearly with input frames. To address this, we propose a Divided Space–Time Mamba block that models intra- and inter-frame long-range dependencies efficiently, resolving scalability issues without sacrificing performance. We refined this approach by introducing a modified Vision Mamba architecture based on a Divided Space–Time Mamba model, as illustrated in Figure 1. By separating Vision Mamba into spatial and temporal modules, the architecture leverages specialized learning for each dimension: the spatial module captures fine-grained details within individual frames, while the temporal module tracks movement and event progression over time. This division is particularly effective for medical video detection, enabling the model to learn dynamic appearance and motion cues more efficiently.

Each frame in the input clip is divided into non-overlapping patches of size P×P. This ensures that the *N* patches cover the entire frame, with *N* defined as $N=HW/P^{2}$. Each token is represented by $x_{\left(p,t\right)}\in R^{3P^{2}}$, where *p* and *t* are spatial locations and a frame index. The sequence of tokens is initially arranged in $X\in R^{N\times T\times D}$, where *N* represents the patch position within each frame, *T* indexes time and *D* is the embedding size of each token.

The encoder blocks process temporal and spatial dimensions separately, one after the other. Each block *l*, we first use $X^{space}\in R^{(B\times T)\times N\times D}$ to fix the temporal dimension. Then, we perform a bidirectional scan across all frames to capture spatial dependencies:

(9) $$\begin{array}{cc} y^{space}\left(t\right) & ={\mathrm{SSM}}_{spatial}\left(X^{space}\left(t\right)\right). \end{array}$$

The output of the temporal B-Mamba scan is then fed forward to compute the temporal B-Mamba encoder where all tokens are grouped based on frames $X^{time}\in R^{(B\times N)\times T\times D}$. The temporal B-Mamba block performs bidirectional selective scans across frames, ensuring that temporal dependencies are aggregated from both past and future contexts. Across both time and space dimensions, separate parameters are learned: $A^{time},B^{time},C^{time}$ for the temporal component and $A^{space},B^{space},C^{space}$ for the spatial component.

### 3.4. Pretraining Approaches

To address the scarcity of labeled data in clinical video settings, we investigated two distinct self-supervised learning (SSL) strategies for initializing our Divided Space–Time Mamba model: a Teacher–Student approach based on semantic distillation, and a fully self-supervised reconstruction strategy using masked autoencoding. The embedded tokens are passed through the encoder and decoder parts, respectively. The encoder part consists of *L* stacked Mamba blocks and aims to extract meaningful latent representations by processing only masked input sequences. These representations capture the context and structure of the visible data while learning to predict missing tokens. In the pretraining stage, the learnable special token is removed.

Teacher-Student SSL: We first attempted a teacher-student SSL approach using CLIP [83] as the teacher model providing semantic guidance. Inspired by previous works [64,81], the decoder part aligns unmasked tokens directly with a linear projection to the teacher model. For masking strategy, we employ a frame-by-frame semantic masking approach, assigning higher probabilities to tokens that carry crucial clues [64,84]. In the PICU setting, this strategy resulted in unstable convergence and poor generalization, which is caused by the semantic gap between the teacher and the target domain and the mismatch between the pretraining objectives. CLIP was trained on generic web images and captions that emphasize everyday objects and scenes, while our clinical data involve subtle and domain-specific patterns such as neonatal anatomy, occlusions, and the presence of medical equipment. The teacher often highlighted irrelevant elements like monitors or cables instead of the anatomical regions required for detection. Additionally, CLIP’s training objective focuses on global image–text alignment, whereas our model requires precise spatial localization and temporal consistency. This misalignment likely led to conflicting gradients during training, especially under high masking ratios and in visually degraded frames.

Masked Autoencoders SSL: UMT relies heavily on the semantic guidance from the teacher model. If this model is pre-trained on general images that are vastly different from PICU environments, the guidance might be less relevant or even misleading. Video Masked Autoencoders’s self-supervised approach allows it to learn directly from the target domain (PICU videos) without relying on potentially mismatched external knowledge. The decoder part consists of stacked B-Mamba blocks with a final output projection to reconstruct the masked video patches. VideoMAE’s masking strategy and reconstruction objective enable it to capture domain-specific features and patterns present in PICU data, even if they’re very different from general image datasets. In the Divided Space–Time Mamba model, and to keep the structure, masked tokens are replaced with learnable parameters. These learnable embeddings act as placeholders for the missing information and are processed alongside the unmasked tokens. The learnable parameters allow gradients to flow through the masked positions, which can improve model optimization.

### 3.5. Depth Information Integration

To enhance the ability of the model to learn subtle anatomical features and motion cues within the complex PICU setting. We augment our Divided Space–Time Mamba architecture by introducing depth maps as an additional input channel alongside RGB frames. For each input video frame $X_{t}\in R^{3\times H\times W}$, we incorporate a corresponding depth map $Dt\in R^{1\times H\times W}$, resulting in a four-channel input $X_{t}^{d}\in R^{4\times H\times W}$. Each token is represented by $x_{\left(p,t\right)}\in R^{4P^{2}}$.

Depth information is fused through early channel-level concatenation prior to tokenization, ensuring pixel-wise alignment between RGB and depth modalities. The fused 4-channel frames are processed by a shared patch-embedding layer and the same Divided Space–Time Mamba encoder, without the use of a separate fusion or attention branch.

During the pretraining phase, we apply the same masking strategies to both RGB and depth channels, encouraging the model to learn the relationships between appearance and geometric features. Our experiments demonstrate that the incorporation of depth information leads to improved performance in both pretraining and downstream tasks, particularly in scenarios requiring precise spatial understanding of the PICU environment.

### 3.6. Fine-Tuning

The pretrained model undergoes fine-tuning on the PICU dataset for face and thoracoabdominal detection. During fine-tuning, the decoder block is replaced with a lightweight detection head comprising three projection layers for classification, bounding box regression, and orientation angle prediction.

The detection task in PICU environments necessitates a multi-component loss function to address distinct challenges inherent to clinical video analysis. The total loss integrates four components:

(10) $$L_{\mathrm{total}}=L_{\mathrm{cls}}+L_{\mathrm{angle}}+\alpha \cdot L_{\mathrm{bbox}}+\beta \cdot L_{\mathrm{IoU}}$$

where α and β balance the contribution of each component based on their typical value ranges during training.

Classification Loss ($L_{\mathrm{cls}}$): Binary Cross-Entropy is employed for object presence prediction. This choice addresses the non-mutually exclusive nature of face and thorax detection in PICU frames, where medical equipment frequently occludes one region while leaving the other visible.

Angle Loss ($L_{\mathrm{angle}}$): Orientation prediction utilizes Cross-Entropy loss with Circular Spatial Layout (CSL), discretizing the 180° rotation space into 180 bins. The CSL formulation addresses the periodicity of angular measurements, where standard Cross-Entropy would incorrectly treat adjacent angles (e.g., 179° and 1°) as maximally different.

Bounding Box Regression ($L_{\mathrm{bbox}}$): L1 loss optimizes the coordinate predictions for bounding box parameters (x,y,w,h). We selected L1 over L2 loss due to its robustness to annotation outliers present in clinical data.

Oriented IoU Loss ($L_{\mathrm{IoU}}$): The rotated Intersection over Union loss directly optimizes the spatial overlap between predicted and ground truth oriented bounding boxes:

(11) $$L_{\mathrm{IoU}}=1-\frac{\mathrm{Area}(B_{\mathrm{pred}}\cap B_{\mathrm{gt}})}{\mathrm{Area}(B_{\mathrm{pred}}\cup B_{\mathrm{gt}})}$$

This component ensures spatial alignment beyond coordinate accuracy, particularly crucial for oriented boxes where axis-aligned IoU would penalize correctly oriented predictions. In cases involving patient rotation, bed tilt, or oblique camera angles, a predicted box may be geometrically correct yet misjudged by axis-aligned IoU metrics due to misalignment with image axes. The rotated IoU (rIoU) metric accounts for both position and orientation, providing a more accurate measure of overlap under rotation. This is particularly important for respiratory motion analysis, where chest orientation must be tracked precisely, and penalizing correctly rotated predictions would compromise model evaluation and training.

## 4. Experimental Protocol

### 4.1. Data Acquisition

To evaluate our approach, we collected two datasets. The first was gathered at the PICU of CHU-Sainte-Justine Hospital (CHU-SJ). To our knowledge, this represents the only available video dataset that captures both facial and thoracoabdominal regions in a PICU setting, as existing datasets typically focus on either face detection or respiratory monitoring in isolation. Due to privacy and ethical constraints, acquiring large-scale annotated PICU video datasets is challenging. Our method overcomes this by generating video-like data from publicly available images, enabling effective pre-training with limited real video data.

#### 4.1.1. CHU-SJ Videos Collection

**Figure 3:**
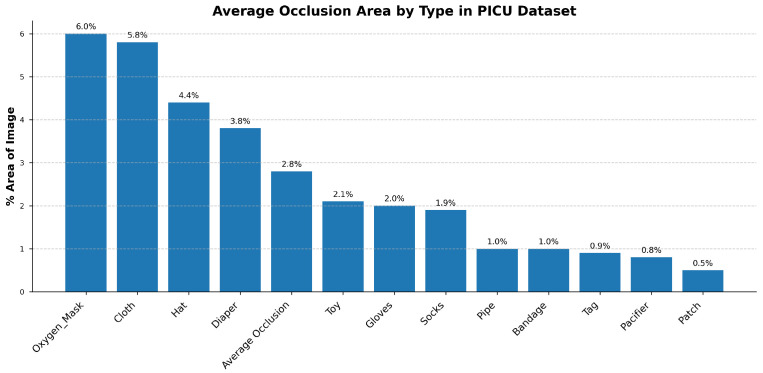
Average image area occluded by common sources in the CHU-SJ PICU dataset. Medical equipment (e.g., oxygen masks, 6.0%) and patient coverings (e.g., cloths, 5.8%) are the dominant occlusion types.

At CHU-Sainte-Justine Hospital’s PICU, videos are collected using a Microsoft Azure RGB-D sensor color camera with 30 FPS (ultra-HD 12-megapixel RGB camera). Approximately 485 different patients admitted to the PICU of CHU-SJ were recorded for 30 s each [85]. Patients, especially infants and young children, frequently moved or shifted positions, causing their faces and thoraxes to move out of the camera’s field of view. Variability in lighting, including low light during nighttime or shadows from medical equipment, significantly affected video quality. The presence of medical devices such as ventilator tubes, masks, or monitoring leads often obscures key regions, complicating the detection process. To quantify these environmental challenges, Figure 3 provides a statistical breakdown of common occlusion sources. As shown, medical necessities like oxygen masks (6.0%) and patient coverings such as cloths (5.8%) and hats (4.4%) are the most significant contributors, underscoring the need for an occlusion-robust detection model.

Faces and thoracoabdominal regions within each video frame were manually annotated using oriented bounding boxes. For face detection, the oriented bounding box was drawn around the face, from the forehead to the chin and from ear to ear. For thoracoabdominal detection, the oriented bounding box covered the area from the upper chest to the diaphragm, including the region of the thorax and abdomen. The dataset comprises individuals with varied attributes, such as skin color, ethnicity, and an age range spanning from 0 to 18 years. This diversity ensures that our dataset captures a wide spectrum of the population across skin tones, genders, and age groups. The institutional ethics committee of Sainte-Justine Hospital approved the study and database construction (protocol code 2016-1242) on 31 March 2016. Prior to video recording, parental consent was obtained by a research assistant trained in human ethics.

#### 4.1.2. Public Data Collection

To address the scarcity of labeled data in medical settings, we constructed a new dataset derived from publicly available images with hospital settings, with a specific focus on neonatal, infant, pediatric, and intensive care units. Approximately 5000 images were collected using these keywords and transformed into video sequences using a domain-specific sequential data augmentation strategy. To simulate the temporal and spatial dynamics of real videos, each image was duplicated into multiple frames, creating the illusion of continuity. Temporal variations, such as adjustments in brightness, contrast, and color, were applied progressively across frames to replicate real-world changes in lighting conditions and image intensity over time. Spatial variations were carefully introduced to enrich the dataset with dynamic visual effects. These included random shifts to simulate minor positional changes, random rotations to emulate natural camera movements, and the addition of subtle noise for enhanced texture realism. Collectively, these augmentations resulted in over 15 k video clips, providing a rich resource for pre-training models. This approach addresses the challenge of limited annotated video data while maintaining high relevance to medical environments, thereby supporting feature learning for our specific environment.

### 4.2. Implementation Details

#### 4.2.1. Pre-Training Stage

We first conduct experiments on the self-supervised pre-training approaches using both datasets simultaneously. Each 30-s patient video is divided into video clips, sampled with a temporal stride of 4 to address the temporal redundancy often present in consecutive frames. Each video clip consists of 16 frames of size 224 × 224. For both SSL pre-training methods, we follow most of the hyperparameter settings described in [9,64], but we use a masking ratio of 80% for both of them. The models are pre-trained on both datasets of 50 k video clips using the AdamW optimizer over 2500 epochs. The training process employs a base learning rate of $1.5\times 10^{-4}$, weight decay of 0.05, and a warm-up period of 40 epochs. We adjust the base learning rate in direct proportion to the total batch size, using the formula lr=base learning rate×batch size/256. The encoder architecture is set to a depth of 12 with an embedding dimension of 768, while the decoder has an embedding dimension of 384. To encourage the student model to learn high-level representations in Teacher-Student SSL, we use Mean Squared Error (MSE) to align unmasked tokens from the student with those from the teacher model. For the reconstruction task in Masked Autoencoders SSL, MSE is applied to measure the difference between the normalized masked pixels and their reconstructed counterparts.

#### 4.2.2. Fine-Tuning Stage

The pre-trained models are fine-tuned on downstream tasks, specifically face and thoracoabdominal detection in the PICU. This evaluation assesses their effectiveness in learning from small, specialized datasets while demonstrating data efficiency, how effectively the model can train on a dataset with a complex setup. We evaluate the model’s data efficiency and transfer learning capabilities through three training scenarios: (1) training from scratch, (2) pre-training on Kinetics-400 followed by fine-tuning on the PICU dataset, and (3) using our VideoMAE pre-trained weights based on Vision Transformer. The fine-tuning architecture consists of a Mamba Encoder backbone with specialized prediction heads for classification, bounding box regression, and rotation angle prediction with 180 categories for each detected object. In order to capture representative frames across the entire video, we employed a uniform sampling strategy with 16 segments per video. For CHU-SJ dataset, we generated 7216 samples, with the first 373 patients used for training and the remaining 112 subjects for testing. We report the results based on the remaining patients. Given the limited dataset size, we additionally performed 5-fold patient-wise cross-validation to verify model stability. Patients were randomly divided into 5 folds ensuring no patient appears in multiple folds. Each fold maintained approximately the same age distribution and occlusion severity. To assess the influence of frame resolution on model performance, we experiment with three resolutions: 224 × 224 and 640 × 640. The training was carried out using the Rectified Adam (RAdam) optimizer, with a learning rate of $1\times 10^{-3}$. Training was conducted for 100 epochs, with a batch size of 32. All experiments were implemented in PyTorch version 2.0.1), and the network was trained on a single NVIDIA Montréal, QC, Canada Tesla V100S-PCIE-32GB GPU.

### 4.3. Evaluation Metrics

#### 4.3.1. Detection Metrics

We evaluate detection performance using mean Average Precision (mAP), following standard protocols adapted for oriented bounding boxes. This includes mAP at specific IoU thresholds of 0.50, 0.60, and 0.75, as well as the comprehensive mAP50-95 metric, which averages performance across IoU thresholds from 0.50 to 0.95. The rotated IoU (rIoU) is computed by determining the exact intersection area of the two convex polygons that define the boxes:

$$\mathrm{rIoU}=\frac{\mathrm{Area}(B_{pred}\cap B_{gt})}{\mathrm{Area}(B_{pred}\cup B_{gt})}$$

where $B_{\mathrm{pred}}$ and $B_{\mathrm{gt}}$ are oriented rectangles parameterized by $\left(x_{c},y_{c},w,h,\theta \right)$.

#### 4.3.2. Temporal Consistency Metrics

Temporal IoU measures detection stability across consecutive frames, quantifying tracking smoothness by averaging the IoU of an object’s bounding box between adjacent frames. A higher value indicates less jitter.

$$\mathrm{Temporal}\mathrm{IoU}=\frac{1}{T-1}\sum_{t=1}^{T-1}\mathrm{IoU}\left(B_{t},B_{t+1}\right)$$

where $B_{t}$ represents the detected bounding box at frame *t*.

#### 4.3.3. Angle Evaluation

Oriented bounding box angles are predicted using Circular Spatial Layout (CSL), which frames the task as a classification problem. We discretize the 180° orientation space into 180 classes, resulting in a 1° resolution. This approach, trained with a Cross-Entropy-based loss, naturally handles the 180-degree periodicity of oriented bounding boxes without discontinuities. Performance is evaluated using Angle Accuracy, defined as the percentage of predictions where the model correctly identifies the exact 1-degree ground truth bin.

## 5. Results

### 5.1. Comparison of Pre-Training Approaches

**Figure 4:**
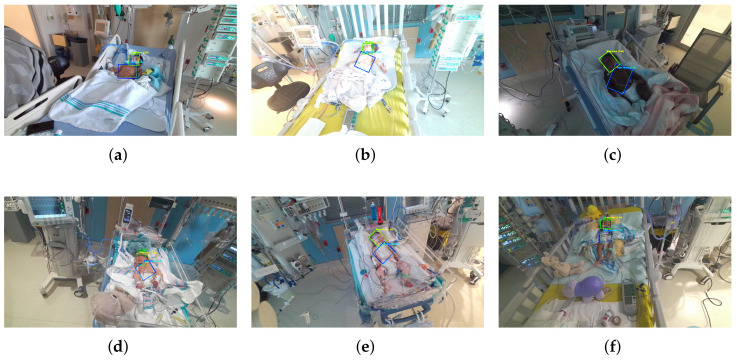
Subfigures (**a**–**f**) illustrate representative PICU frames from different patients under varying levels of occlusion and lighting conditions. Solid lines indicate ground truth annotations (green: face, blue: thorax), and dashed lines represent model predictions (cyan: face, yellow: thorax).

Figure 4 presents qualitative results comparing predicted outputs with ground truth annotations for representative frames, demonstrating the model’s ability to detect and localize regions of interest accurately.

The comparative results of various pre-training strategies highlight the challenges of applying them in specialized domains such as the PICU, where data scarcity and distinct visual characteristics complicate transfer learning. Pediatric data differs significantly from adult datasets, and the presence of uncontrolled lighting, occlusions from medical equipment, varying poses, and intra-domain variability exacerbates the difficulty of learning robust representations. As shown in Table 1, models trained from scratch or fine-tuned from supervised pre-training on Kinetics-400 struggle to converge effectively in this setting. Scratch training suffers from overfitting risks due to the simultaneous need to learn both low-level and domain-specific features, while Kinetics-400 pre-training, designed for action recognition, offers limited benefit for fine-grained spatial tasks like face and thoracoabdominal detection.

**Figure 5:**
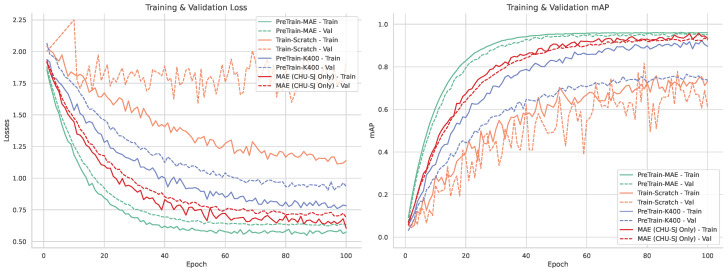
Comparison of training strategies. The PreTrain-MAE model, which was pre-trained using an augmented dataset of video clips generated from real clinical images, shows faster convergence and achieves a higher final mAP compared to the other baselines.

This performance gap is reflected in the training and validation curves shown in Figure 5. The scratch-trained model shows slow mAP improvement and high variance, while the Kinetics-400 pre-trained model converges faster but fails to generalize well due to domain mismatch. In contrast, masked self-supervised pre-training achieves smoother loss curves and consistently better mAP, indicating stronger domain alignment and robustness to PICU-specific challenges.

While the Teacher–Student paradigm (UMT) outperforms both baseline models, its reliance on a CLIP-based teacher, which is trained on generic image distributions, limits its effectiveness in medical settings. In the PICU, where scenes often include tubes, blankets, and neonatal anatomy, this external guidance can introduce noise, misdirecting the student and destabilizing convergence. As shown in Table 1, UMT achieves only 0.50 mAP@0.75, compared to 0.70 for the Masked Autoencoder (MAE). Unlike UMT, MAE operates without an external teacher and learns directly from the target data through high-ratio tube masking. This purely self-supervised strategy captures subtle appearance cues and motion patterns intrinsic to the PICU, enabling higher rIoU, mAP, and angle accuracy. Overall, MAE’s domain-native learning approach proves more effective and reliable in clinical video environments characterized by data scarcity and complexity.

Furthermore, the effectiveness of our self-supervised approach is significantly enhanced by our data augmentation strategy. As illustrated in Figure 5, a direct comparison between the model pre-trained on clinical data alone (MAE (CHU-SJ Only)) and the one augmented with augmented clips (PreTrain-MAE) reveals a substantial performance gain. The model leveraging augmented sequences derived from real clinical data not only converges significantly faster but also achieves a higher final mAP and a lower training loss. This result, quantified in Table 1, provides direct evidence that our augmented data generation is a key contributor to the model’s success, serving as an effective method to overcome data scarcity and improve generalization in this challenging clinical domain.

### 5.2. Model Validation

To verify the robustness of our best-performing model, we conducted 5-fold patient-wise cross-validation using the 373 training patients. The 112 test patients were held out exclusively for final evaluation and were not included in cross-validation. Patients were randomly divided into 5 folds of approximately 74–75 patients each, ensuring no patient appeared in multiple folds. Each fold maintained similar distributions of age groups, occlusion severity, and recording conditions to avoid bias.

The cross-validation results in Table 2 demonstrate highly consistent performance across all folds, with minimal variance in key metrics (mAP@0.5: σ = 0.006, rIoU: σ = 0.005). This low variability indicates that our model generalizes well across different patient populations and is not overfitting to specific patient characteristics.

### 5.3. Comparison with State-of-the-Art Methods

To evaluate the effectiveness of our DST-Mamba approach, we compared it against established frame-based and video-based detection models, as summarized in Table 3. Frame-based models such as YOLOv8-m achieve high single-frame accuracy (0.892 mAP) but lack temporal coherence, resulting in unstable region-of-interest (ROI) tracking and jitter, which negatively impacts physiological signal estimation. This instability introduces motion artifacts into pixel-level signals, degrading the accuracy of downstream heart rate and respiratory estimation pipelines, particularly in neonates with subtle physiological cues. Furthermore, applying YOLOv8 across 16 frames incurs substantial computational load (634.6 GFLOPs) and high latency (243 ms). Incorporating DeepSORT [14] improves temporal consistency (0.82 temporal IoU) but further increases inference time. ViTDet [32] was also benchmarked in a per-frame configuration. While it achieved reasonable localization performance (0.60 mAP50-95), its high latency (115 ms) and parameter count (102M) reduce its suitability for real-time clinical deployment.

In contrast, video-based models such as I3D-FPN [20] and TimeSformer [17] either suffer from low detection accuracy (e.g., 0.360 mAP for I3D-FPN) or require significantly more parameters (up to 121M for TimeSformer) while still falling short in temporal stability. VideoMAE (ViT-B) [9] achieves stronger performance (0.920 mAP, 0.90 temporal IoU) but with higher memory requirements and moderate latency. Notably, VideoMamba [81] achieves competitive performance across the board (0.940 mAP, 0.420 mAP50-95, 0.92 temporal IoU) while maintaining the lowest GFLOPs (5.71) and latency (35 ms), making it a strong benchmark for efficiency.

Our proposed DST-Mamba model directly addresses this challenge by achieving the highest detection accuracy (0.960 mAP) and the highest localization precision, with a 0.620 mAP50-95. Despite a modest increase in GFLOPs (7.56) and latency (43 ms) compared to VideoMamba, DST-Mamba offers a better overall trade-off between efficiency and accuracy. This advantage stems from its Divided Space–Time architecture, which enables specialized spatial and temporal learning without the overhead of joint attention. These findings suggest DST-Mamba is potentially suitable for real-time processing, pending clinical validation, balancing accuracy, stability, and efficiency critical for downstream physiological signal extraction.

### 5.4. Ablation Studies

#### 5.4.1. Space–Time Mamba Architecture

We compared our sequential Mamba architecture with joint and parallel design variants, as shown in Table 4. While the joint model demonstrated slightly better computational efficiency (1.39 GFLOPs, 35 ms), it yielded lower detection accuracy (0.91 mAP). In contrast, the parallel architecture exhibited a severe performance drop (0.31 mAP), clearly indicating that independently modeling spatial and temporal features is insufficient for accurate region-of-interest (ROI) detection in PICU environments.

**Figure 6:**
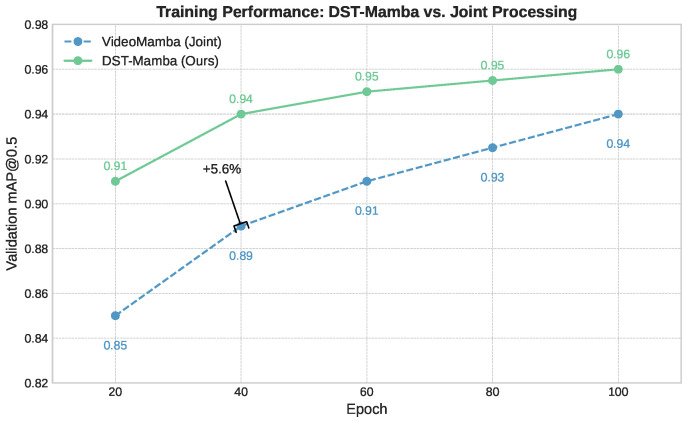
Training progression of DST-Mamba compared to the joint processing baseline. DST-Mamba achieves smoother convergence and consistently higher mAP.

Our proposed sequential design achieves the best balance, delivering the highest accuracy (0.95 mAP) with only a modest increase in computational cost (1.85 GFLOPs, 43 ms). These results strongly support our hierarchical design choice: extracting spatial features first, followed by temporal modeling, leads to more robust and reliable detection in complex clinical video settings. As illustrated in Figure 6, DST-Mamba converges faster and more smoothly than joint processing, ultimately reaching higher accuracy.

#### 5.4.2. Comparative Analysis of Model Architectures

Table 5 compares the trade-off between computational efficiency and detection accuracy across three video models. While ViT achieves strong performance (0.91–0.95 mAP), it incurs significantly higher computational costs, ranging from 50.92 to 415.71 GFLOPs, and has a large parameter count (86.23M). In contrast, VideoMamba delivers competitive results (0.90–0.94 mAP) with substantially lower FLOPs (0.69–5.71) and a smaller model size (54.14 M parameters). Notably, the Divided Space–Time Mamba model achieves the highest accuracy (0.96 mAP) at 16 frames and 640^2^ resolution, while maintaining a moderate computational footprint (7.56 GFLOPs). This result highlights the effectiveness of its factorized design in achieving a favorable balance between accuracy and efficiency.

#### 5.4.3. Model Components

The results in Appendix A Table A1 demonstrate the impact of different architectural components on detection performance. Removing the angle loss function (Without Angle Loss) leads to the lowest performance across all metrics (IoU = 0.81, mAP@0.50 = 0.33, MAE = 0.37), confirming the importance of angle supervision for accurate localization and orientation. The fixed-angle variant (Without Orientation), which assumes vertical alignment, achieves high IoU (0.92) and zero angle error by design, but only moderate mAP (0.487), indicating limited adaptability to real-world orientation variability. Omitting the rotated IoU (Without rIoU) preserves high IoU (0.90) and mAP (0.92), but yields poor angular precision (MAE = 0.40), underscoring the importance of including orientation-aware overlap metrics. The depth-enabled model (With Depth) achieves the highest IoU (0.96) and mAP@0.50 (0.95), though with a slightly elevated MAE (0.52), suggesting that while depth improves spatial localization, it may increase complexity in estimating precise object orientation. These results underscore the necessity of integrating angle loss, orientation modeling, and rotated IoU, alongside depth information, for robust detection in complex clinical scenes.

#### 5.4.4. Pre-Training Masking Ratio

**Figure 7:**
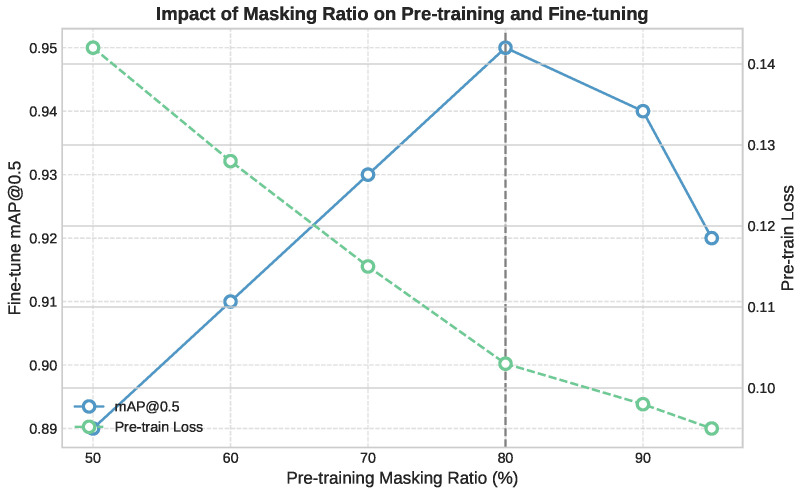
Impact of pre-training masking ratio on downstream detection performance. An 80% masking ratio achieves the lowest pre-training loss and highest mAP@0.5.

In Appendix A Table A2 shows how different masking ratios affect both the efficiency of self-supervised pre-training and the performance on the downstream detection task. Among the configurations tested, a ratio of 80% provides the best trade-off: it achieves the highest mAP@0.5 (0.95), requires fewer fine-tuning epochs (70), and significantly reduces GPU memory usage (7.73 GB). This ratio introduces enough reconstruction difficulty to promote strong representation learning while preserving sufficient spatial context. In contrast, higher masking ratios (90–95%) reduce memory even further but slightly degrade accuracy, likely due to excessive information removal during pre-training. On the other end, lower ratios (50–70%) offer more visual cues but result in higher memory usage and slower convergence. These results confirm that 80% masking provides the best balance for our setting, optimizing resource usage while improving both learning speed and final detection accuracy. Figure 7 shows how different masking ratios affect pre-training and downstream detection. An 80% masking ratio provides the best trade-off, achieving the lowest pre-training loss and the highest fine-tuned mAP@0.5, while also reducing GPU memory usage and convergence time.

#### 5.4.5. Model Depth and Qualitative Results

Table A3 in Appendix A summarizes the impact of encoder depth on model performance and computational efficiency. Increasing the number of layers from 4 to 12 leads to a substantial improvement in mAP@0.50 (from 0.88 to 0.95) and rIoU (from 0.90 to 0.96). However, further increasing the depth to 16 layers provides only marginal gains (0.96 mAP@0.50) while significantly increasing parameters and FLOPs. Thus, the 12-layer configuration (73.7 M parameters, 1.85 GFLOPs) offers the best trade-off between accuracy and efficiency.

### 5.5. Robustness to Clinical Occlusions

To better understand the limitations of our DST-Mamba model in clinical practice, we conducted a detailed error analysis across varying occlusion conditions. Test set predictions were manually categorized into four occlusion severity levels based on the percentage of anatomical region visibility, as shown in Appendix A Table A4. For the None category (less than 10% occlusion), the model achieved IoU of 0.96 and mAP@0.50 of 0.98, demonstrating robust performance under ideal visibility conditions. Light occlusion (10–25%), typically caused by medical tubes or monitoring leads, resulted in minimal performance degradation with an IoU of 0.95 and a mAP@0.50 of 0.96. Moderate occlusion (25–70%), including scenarios where blankets or oxygen masks partially covered the regions of interest, showed more substantial impact with IoU dropping to 0.88 and mAP@0.50 to 0.89. The mAP50-95 metric decreased from 0.61 to 0.52 between light and moderate occlusion levels, indicating reduced localization precision at higher IoU thresholds. Severe occlusion (exceeding 70% coverage) represented the primary failure mode for our model. Performance degraded significantly with an IoU of 0.61 and a mAP@0.50 of 0.58. The mAP50-95 dropped to 0.31, reflecting poor localization accuracy across all IoU thresholds. The 0.27 IoU drop from moderate to severe occlusion indicates substantial detection instability when most of the target region is obscured. These results demonstrate that while DST-Mamba maintains acceptable performance under partial occlusion, severe occlusion remains a significant challenge.

## 6. Discussion

**Figure A1:**
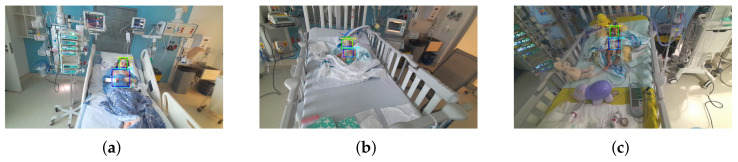
Representative failure cases under challenging conditions. (**a**) partial patient occlusion of the face or thoracoabdominal region by medical equipment or coverings. (**b**) severe occlusion from medical devices. (**c**) low-illumination scene.

Our results indicate that the Divided Space–Time (DST) Mamba architecture directly addresses the core obstacles of PICU video detection, setting a new state of the art for face and thoracoabdominal localization. By factorizing spatiotemporal modeling space-to-time, the model preserves temporal dynamics under motion and occlusions, while oriented boxes (OBBs) with circular smooth label (CSL) supervision explicitly handle orientation variability and camera skew, improving rIoU and reducing angle error. Domain-native self-supervised pretraining (MAE/UMT) mitigates data scarcity and domain shift, yielding higher mAP and more reliable convergence than training from scratch or Kinetics-400 finetuning. Integrating depth (RGB–D) further improves localization in occlusion-heavy, low-contrast scenes, with a measured compute trade-off and occasional angle-error increase that we report. Finally, the state-space formulation confers linear-time complexity and real-time, deployment-oriented efficiency (e.g., ∼23 FPS at $16\times 640^{2}$ with ∼7.56 GFLOPs) while outperforming strong frame-wise and video baselines on accuracy and stability. Representative failure cases are presented in Appendix A Figure A1, illustrating reduced detection accuracy under severe occlusion, low illumination, and partial patient rotation.

### 6.1. From High-Accuracy Detection to Clinical Reliability

The extraction of physiological signals using non-contact methods like remote photoplethysmography (rPPG) is highly sensitive to the stability and consistency of the input ROI. Frame-based detectors process each frame independently, which introduces spatiotemporal jitter and increases inference time, limiting real-time applicability in clinical settings. In contrast, our DST-Mamba model achieves a high temporal IoU of 0.95, ensuring temporally coherent and stable ROIs. This stability reduces non-physiological motion noise and improves the signal-to-noise ratio (SNR), which is essential for reliable physiological monitoring. A mean Average Precision of 0.96 reflects both technical accuracy and clinically meaningful consistency in anatomical localization across frames. This level of performance minimizes gaps in region tracking, reducing the risk of signal disruption during rPPG or respiratory extraction. In critical care settings, even short detection lapses can lead to missed events or delayed alerts, making stable and accurate detection essential for continuous, high-fidelity monitoring. Additionally, the high rotational IoU ensures precise anatomical localization, preventing signal contamination from surrounding regions and reducing the risk of false alarms in intensive care environments.

### 6.2. Limitations & Future Work

The results of this study must be considered in the context of several key limitations. First, the model was developed and validated using data from a single institution. Its performance on data from other PICUs, which may differ in lighting conditions, camera configurations, and clinical protocols, remains untested. To our knowledge, no other publicly available PICU video datasets exist that capture both facial and thoracoabdominal regions simultaneously, which necessitated our reliance on single-center data. To address potential site-specific bias, our framework integrates multimodal RGB–Depth inputs, which are inherently less sensitive to lighting variations and camera-specific color calibration. In addition, we perform self-supervised pre-training on a heterogeneous corpus of over 50,000 video clips comprising both real PICU recordings and synthetically generated hospital scenarios with diverse illumination, contrast, and viewpoints. This domain-diverse pretraining strategy serves as an implicit form of cross-institutional regularization and enhances the model’s robustness to unseen acquisition conditions. A multi-center validation is therefore a critical next step to assess whether the model can generalize. Although this study incorporates depth maps during training, real-time depth capture is not yet standard in most PICU monitoring systems. Future work should assess the feasibility and clinical value of integrating low-cost depth sensors at the bedside to enable robust 4D video analysis.

Second, the dataset is limited to 485 patients due to the practical and ethical challenges of data acquisition in pediatric critical care. The absence of public video datasets for this task necessitated our use of a synthetic data generation strategy. This strategy, while beneficial to pre-training performance in our experiments, is itself a limitation. We acknowledge that this method primarily provides rich spatial augmentation and does not capture true, physiologically relevant temporal dynamics. However, our results demonstrate that this spatial pre-training provides a crucial foundation, allowing the model to generalize much more effectively when subsequently fine-tuned on real clinical videos where it learns the relevant temporal patterns. Further work is required to determine if this method captures meaningful temporal dynamics or primarily provides spatial augmentation. Third, this study’s scope is confined to the detection and tracking of anatomical regions. The work does not validate whether the improved detection metrics translate to more accurate downstream vital sign extraction. Establishing this link between technical performance and clinical utility is a crucial future step.

Finally, the model has not been tested in a live clinical workflow. Any claims regarding deployment readiness are premature, as real-world use requires prospective testing, integration with hospital IT systems, and navigating regulatory pathways.

Future work involves integrating our DST-Mamba architecture with vital sign extraction algorithms for prospective clinical validation against contact monitors. To address data scarcity and foster collaboration, we will release our open-source code and augmented data generation methodology upon publication. The code will be made publicly available at: https://github.com/mkbensalah/Divided-Space-Time-Mamba, accessed on 29 October 2025.

## 7. Conclusions

In this paper, we presented the Divided Space–Time Video Mamba framework for medical video detection in Pediatric Intensive Care Unit (PICU) environments. By decoupling spatial and temporal processing, our approach achieves high accuracy (0.95 mAP@0.50) while maintaining computational efficiency. The incorporation of masked autoencoder pre-training further improves performance, reaching 0.96 rIoU and 0.95 mAP@0.50, and shows improved performance on our single-center dataset. Additionally, integrating depth information enhances the model’s robustness to occlusions and variable lighting conditions. The efficiency of DST-Mamba supports its integration into real-time clinical systems. With 7.56 GFLOPs and 73M parameters, the model processes 16-frame inputs at 640 × 640 resolution in 43 ms, achieving 23 FPS. Its linear complexity ensures predictable scalability with respect to input resolution and sequence length, unlike transformer-based models that scale quadratically. This makes it suitable for deployment on edge devices such as bedside monitors or portable diagnostic tools in the PICU. Furthermore, the divided space–time structure promotes interpretability by isolating spatial and temporal contributions. This separation facilitates the analysis of detection failures and helps verify consistency across frames. The use of oriented bounding boxes produces rotation-aware outputs that align with clinical requirements, especially in respiratory monitoring where thoracic orientation directly influences signal quality. In addition, saliency-based visualizations or attribution maps can be employed to reveal the specific regions within each frame that most influence the model’s predictions. Such visual feedback allows clinicians to verify that the model attends to relevant anatomical features, rather than being misdirected by medical equipment, patient coverings, or shadows. Future work will focus on extending DST-Mamba toward cross-device generalization by evaluating its robustness across different RGB-D sensors and acquisition settings, and by incorporating domain-adaptation techniques to mitigate sensor-specific variability. We will also investigate lightweight pruning, quantization, and token-reduction strategies to enable efficient deployment on embedded and bedside monitoring systems. This study was conducted under institutional ethical approval from CHU Sainte-Justine, with all video data processed within secure research servers. Future work will explore privacy-preserving edge AI and federated learning frameworks to ensure patient data remain local while enabling continuous, on-device model adaptation for real-time bedside use.

## Figures and Tables

**Table 1:** Performance comparison of pre-training strategies using rotated IoU (rIoU), mean Average Precision (mAP) at different thresholds, and angle accuracy. The best results are bolded.

Model	rIoU	mAP@0.50	mAP@0.60	mAP@0.75	Angle
From Scratch	0.85	0.56	0.41	0.22	0.25
K400	0.88	0.61	0.44	0.24	0.28
Teacher-Student	0.95	0.92	0.76	0.50	0.35
MAE (CHU-SJ Only)	0.94	0.94	0.82	0.65	0.37
PreTrain-MAE (Synth.)	**0.96**	**0.95**	**0.85**	**0.70**	**0.40**

**Table 2:** Five-fold patient-wise cross-validation results for DST-Mamba model demonstrating stability across different patient subsets.

Fold	Train/Test	mAP@0.5	mAP@0.75	rIoU	Angle MAE	Temporal IoU
1	298/75	0.948	0.682	0.952	0.41	0.94
2	299/74	0.962	0.705	0.961	0.39	0.96
3	298/75	0.951	0.693	0.958	0.42	0.95
4	300/73	0.957	0.701	0.963	0.40	0.95
5	297/76	0.960	0.698	0.955	0.38	0.94
Mean	-	0.956	0.696	0.958	0.40	0.95
±SD	-	±0.006	±0.009	±0.005	±0.015	±0.008
95% CI	-	(0.949, 0.963)	(0.685, 0.707)	(0.952, 0.964)	(0.382, 0.418)	(0.940, 0.960)

**Table 3:** Comprehensive comparison of models on accuracy, efficiency, and temporal stability for a 16-frame sequence.

Model	GFLOPs	Params (M)	Latency (ms)	mAP@0.5	mAP50-95	Temporal IoU
Frame-based Models						
YOLOv8-m	634.6	26	243	0.892	0.445	0.75
YOLOv8-m + DeepSORT	634.6	28	352	0.926	0.465	0.82
ViTDet (per-frame)	270	102	115	0.69	0.60	0.80
Video-based Models						
I3D-FPN	174.38	35	180	0.360	0.200	0.50
TimeSformer	380	121	75	0.785	0.240	0.65
VideoMAE (ViT-B)	101.9	86	83	0.920	0.330	0.90
VideoMamba	5.71	54	35	0.940	0.420	0.92
**DST-Mamba (Ours)**	**7.56**	**73**	**43**	**0.960**	**0.620**	**0.95**

**Table 4:** Ablation study on Mamba-based architectures. Performance is evaluated for a 16-frame sequence at 224 × 224 resolution.

Model Variant	GFLOPs	Params (M)	Latency (ms)	mAP@0.5
Parallel Space–Time	1.92	81	90	0.31
Joint Space–Time	1.39	54	35	0.91
**Sequential (Ours)**	**1.85**	**73**	**43**	**0.95**

**Table 5:** Model performance and efficiency comparison. This table details the computational cost (FLOPs), model size (Parameters), and mean Average Precision (mAP) for different architectures, input resolutions, and frame counts.

Model	Frames	Input Size	FLOPs (G)	Parameters (M)	mAP
ViT	8	$224^{2}$	50.92	86.23	0.91
ViT	16	$224^{2}$	101.85	86.23	0.93
ViT	16	$640^{2}$	415.71	86.23	0.95
VideoMamba	8	$224^{2}$	0.69	54.14	0.90
VideoMamba	16	$224^{2}$	1.39	54.14	0.91
VideoMamba	16	$640^{2}$	5.71	54.14	0.94
Divided Space–Time	8	$224^{2}$	0.93	73.65	0.89
Divided Space–Time	16	$224^{2}$	1.85	73.65	0.95
Divided Space–Time	16	$640^{2}$	7.56	73.65	**0.96**

## Data Availability

The clinical video data supporting this study are from the MEDEVAC database at CHU Sainte-Justine Hospital, collected under ethics protocol 2016-1242 (approved 31 March 2016). These data cannot be made publicly available due to ethical and privacy restrictions protecting pediatric intensive care patients. The database infrastructure is described in Boivin et al., Sensors 2023, 23, 5293 [85]. Access is subject to Research Ethics Board approval, and interested researchers may contact the corresponding author for further information. The augmented video clips were generated from publicly available images using our methodology. Code and augmentation methodology will be available upon publication.

## Appendix A. Tables

**Table A1:** Ablation study on model components. This table shows the impact of removing or adding specific features, such as angle loss, fixed orientation, rotated IoU (rIoU), and depth information, on overall detection performance.

Model Variant	IoU	mAP@0.50	Angle
Without Angle Loss	0.81	0.33	0.37
Without Orientation	0.92	0.487	0.00
Without rIoU	0.90	0.92	0.40
With Depth	**0.96**	**0.95**	0.52

**Table A2:** Impact of masking ratio on self-supervised pre-training efficiency and downstream task performance.

Masking Ratio	Fine-Tune Epochs	mAP@0.5	Memory (GB)
50%	95	0.89	12.28
60%	85	0.91	10.73
70%	80	0.93	9.22
**80%**	**70**	**0.95**	**7.73**
90%	70	0.94	6.21
95%	75	0.92	5.45

**Table A3:** Impact of Mamba encoder depth on model performance and efficiency. All experiments use 224 × 224 input resolution with 16-frame clips. The optimal configuration is bolded.

Layers	Parameters (M)	FLOPs (G)	mAP@0.50	rIoU
4	43.2	0.85	0.88	0.90
8	53.1	1.15	0.92	0.93
12	**73.7**	**1.85**	**0.95**	**0.96**
16	96.4	2.45	0.96	0.97

**Table A4:** Performance across occlusion levels.

Occlusion Level	IoU	mAP@0.50	mAP50-95
None	0.96	0.98	0.68
Light	0.95	0.96	0.61
Moderate	0.88	0.89	0.52
Severe	0.61	0.58	0.31
